# Hatchlings and Neonate Turtle Gonads Have Spatially Restricted Neural Processes

**DOI:** 10.17912/micropub.biology.001327

**Published:** 2025-04-15

**Authors:** Jeanette Wyneken, Boris M. Tezak, Debra Lee Miller

**Affiliations:** 1 Biological Sciences, Florida Atlantic University, Boca Raton, Florida, United States; 2 Department of Cell Biology, Duke University, School of Medicine, Durham, North Carolina, United States; 3 Department of Biology, Wesleyan University, Middletown, Connecticut, United States; 4 Center for Wildlife Health (FWF) , University of Tennessee at Knoxville, Knoxville, Tennessee, United States

## Abstract

Morphological and molecular evidence explains the lack of nociception (“pain”) associated with very small, laparoscopic gonadal biopsy in neonate turtles. This safe procedure serves to verify neonate sex of late-maturing species, such as sea turtles. Ethical concerns about the potential for biopsy pain, inferred from mammals, limited access to sex verification biopsy for decades. Yet, standard behavioral evidence of pain during biopsy (e.g., escape attempts, biting, guarding behavior after biopsy, inappetence) were negative. Morphological and molecular evidence early in ontogeny, shows that, unlike mice, young turtles have limited neural processes to the gonadal medulla and none reach the cortical layer.

**Figure 1. Turtle gonad study panels and controls:**
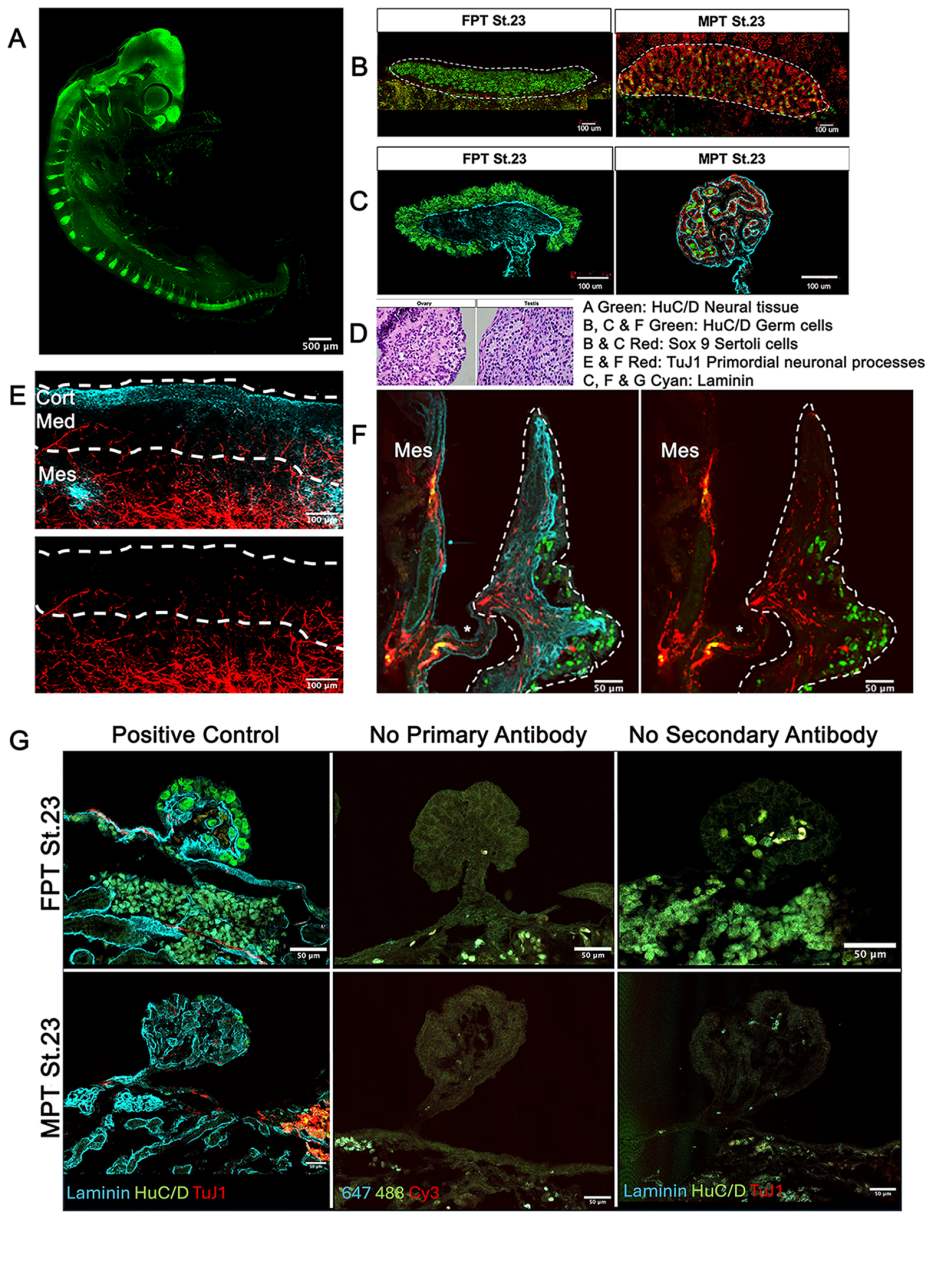
(A) Red eared slider turtle ( *T. scripta* ) embryo (St.12) labeled with HuC/D (green) shows that HuC/D reliably labels developing neural tissue in the CNS and early PNS; note the neural tube, sensory vesicles, prominent peripheral nerve roots, and the linear mesonephros are bright green. (B) Representative images of St.26 *T. scripta* ovary and testis whole mounts labeled with HuC/D (green) and show elliptical germ cells (GCs) labeled green; many are in the ovary and very few are in the testis. The Sox9 (red-labeled) supportive (Sertoli) cells dominate the testis. Dashed lines outline the gonad (Cort: cortex, Med: medulla; Mes: Mesonephros). (C) Cross sections of St.26 *T. scripta* ovary and testis that identify the elliptical germ cells are isolated in the cortex of both males and females. Laminin (cyan), is a key part of the basement membrane. Based on the location and morphology of the cells labeled with HuC/D, we determined that all HuC/D positive cells observed in these samples were GCs and not neural bodies. Note that HuC/D labeling in the germ cells is distinct and remote from the labeling observed in the neural bodies. (D) Neonate *C. caretta* ovary (left) and testis (right) sections stained with H&E provide clear structural comparisons between the multicellular cortex of the ovary overlying a “disorganized” medulla. In contrast, the testis has a single-cell layered cortex (= “tunica”) overlying organized medullary cords, photographed at 400X. (E) Representative sagittal series image of optical Z-sections of neonate *C. caretta* ovary wholemount labeled with TuJ1 (red) for neural processes and Laminin (cyan) showing the distinct neural processes and a few neural bodies concentrated in the mesonephros. Note that few processes extend into the medulla, and none in the cortex. Dashed lines outline the ovary. No *C. caretta* testis was available due to high nest temperatures. (F) Representative cross-sections of a neonate *C. caretta* ovary, mesovarium, and mesonephros are labeled with HuC/D, Cyan, and TuJ1 (red). Mesonephros (Mes) and mesovarium (under the *), have positive neural processes. The roughly triangular gonad, toward the right in each panel, has sparse positive neural projections that are restricted to the ovarian medulla and do not reach the outer (cortical) domain. Neural processes are filamentous whereas germ cells are roughly elliptical. The HuC/D (green) labeled GCs in the ovarian cortex are similar to the *T. scripta* samples. Note, there are no signs of HuC/D positive neural bodies in the gonad. The presence of Tuj1 projections does not identify the type of neuron (sensory, motor, or interneuron) that might form later in ontogeny. (E-F) Gonads are outlined in white dashed lines. (G) Positive control and antibody labeling controls (primary and secondary) for fluorescent antibody labeling of *T. scripta* ovaries (upper row) and testis (lower row). Middle panel, testis 647, 488, and Cy3 identify the wavelengths excited by the fluorescent labels. FPT: female promoting incubation temperatures; MPT: male promoting incubation temperatures; St: developmental stage.

## Description

Most turtles, like many other reptiles, lack heteromorphic sex chromosomes and are not sexually dimorphic until puberty (Janzen and Phillips 2006). Understanding that sex-specific morphogenesis is directed by incubation temperatures in most turtles has been known for 4-5 decades (Pieau 1972, Yntema 1976, Yntema 1979, Mrosovsky and Yntema 1980, Yntema and Mrosovsky 1980, Wibbels et al. 1998). Testes and ovaries differentiate morphologically from a bipotential gonad precursor during the middle of embryonic development, yet external sexual dimorphism occurs well after hatching – years (many freshwater turtles) to decades (cheloniids) later. Sex identification of hatchlings or neonates is important for establishing baseline sex ratios and thus, for identifying sex ratio shifts over time.

Incubation temperature is the most important factor driving sex differentiation, yet specific associations between incubation temperatures and hatchling sex remain elusive except at the warmest and coolest temperatures that are compatible with embryonic growth. Sex identification of hatchlings or neonates is important for establishing baseline sex ratios and, thus, identifying shifts in sex ratios over time. Sex ratios are fundamental to population dynamics. Carter & Hopkins (2022) summarize the most fundamental importance of knowing sex ratios and their contexts. Hartl (1988) reminds us that sex ratio is a linchpin of population dynamics and conservation research. Others clearly note that sex ratios are central in conservation research (Hendry et al., 2017; Geffroy and Wedekind, 2020, 2021). For threatened and endangered species, conservation management necessarily relies on the best available data. Due to the highly imperiled status of many chelonian species, and the delayed age to maturity of many species, accurate demographic metrics are important to conservation efforts (Rhodin et al., 2018, Stanford et al., 2020).

*Importance of sex identification* . The species examined here have a warm female/cool male sex determining system yet estimating sex ratios across populations is challenging. Sex identification in neonate turtles has become increasingly important due to changing climatic conditions. Very few studies verify the sexes of their hatchlings, instead relying on proxies, many of which are based upon erroneous assumptions regarding their accuracy (Girondot et al., 2010, Wyneken and Lolavar 2015). There are two methods providing histological verification: hatchling sacrifice (Godfrey et al., 1999, Mrosovsky and Yntema 1980, Miller and Limpus 2003) or laparoscopic sex identification with biopsy verification (Wyneken et al. 2007). Hatchling sacrifice is 100% accurate yet often not permitted with imperiled species. Laparoscopic sex identification is low impact, yet requires rearing the turtles for a few months, allowing for sufficient yolk sac depletion to permit the telescope room to focus. For each species, gonadal biopsy for histologic verification is necessary to verify that the sex identification criteria used are correct across seasons and clutches (Wyneken et al., 2007).

However, ethical concerns about the biopsy producing “pain” severely limited access to sex verification biopsy for almost 5 decades. The assumption that this procedure produces pain is primarily based on work with mammals (mainly mice) in which the gonads are innervated during embryonic development. Yet, it is important to note, that the presence of receptors specifically activated by painful stimuli (nociceptors) in gonads has not been studied and that the presence of neural projections in a tissue does not indicate nociception (Steeds 2009).

*Turtle gonadal development is not like that of mammals* . McKey et al. (2019) demonstrated that in mouse ( *Mus musculus* ), peripheral innervation is involved in mammalian sex-specific gonadal developmental patterning. Neural crest-derived neurons invade the dorsal surface of the mouse ovary during embryonic development and give rise to a neural network, whereas in male mice, the epididymis and vas deferens receive innervation, but the body of the testis does not.

In the present study, we used similar immunofluorescent labeling and imaging protocols as McKey et al., (2019) to examine turtle gonads for innervation. Our work verified that immunofluorescent labeling for HuC/D (RNA binding proteins essential for neuronal differentiation) reliably labels nervous tissue in turtles prior to hatching and we found no signs of HuC/D positive neural bodies in the developing ovaries or testes ( *T. scripta* ) turtles ( Figure 1A- C, ).

Separately, 123 neonate sea turtle biopsy samples ( *C. caretta* [Wyneken et al 2007] and *Chelonia mydas* [unpublished QA/QC for Wyneken and Lolavar 2015]) were fixed in 10% buffered formalin and prepared as 3-5 um thin sections. These were H&E counterstained following standard procedures outlined by Humason (1979, pp 118-120). H&E histology slides were examined and no nervous tissue was found (Fig 1D). If peripheral nerves were present, as in mammals, they would appear as wavey bundles of tubes encased in fine connective tissue.

The immunofluorescence analysis of *C. caretta* neonate ovaries labeled for HuC/D and the pan-neuronal marker TUJ1 support the hypothesis that HuC/D positive neural bodies are absent from the turtle gonad and reveal the presence of few neuronal projections that invade the ovarian medulla via the mesovarium (Fig 1 E,F). Interestingly, these TuJ1 positive innervations do not extend into the ovarian cortex. Altogether, unlike what is observed in mice, our work described here suggests that turtle gonadal developmental patterning is independent of innervation. Turtle ovaries and testes begin to differentiate during the middle third of development, yet no innervation of the gonads is observed during this period. Additionally, we found no evidence of neural bodies inside turtle gonads throughout development and none in neonate ovaries but confirm that there is some level of neuronal projections reaching the ovarian medulla in *C. caretta* neonates. Importantly, the presence of TuJ1 projections should not be inferred to be sensory receptors (or nociceptors), rather they may serve in defining structure in these very young animals.

*Why does gonadal innervation matter?*

The prohibition of laparoscopic gonadal biopsy for histological sex verification in neonate sea turtles reduces the accuracy of this nonlethal sex identification tool. Consequently, a very small subset of users can reliably sex neonates and new personnel cannot verify their accuracy. Further, new, less invasive molecular techniques are stalled by limited verifications. Yet, turtle conservation management quality is based upon understanding of sex ratios, which are essential years before the turtles reach maturity (Wyneken et al., 2007, Wyneken and Lolavar 2015).

## Methods

H&E histology slides were examined for nervous tissue. No nervous tissue was found (Fig 1D). Separately, 123 neonate sea turtle biopsy samples ( *C. caretta* [Wyneken et al 2007] and *Chelonia mydas* [unpublished QA/QC for Wyneken and Lolavar 2015]) were fixed in 10% buffered formalin and prepared as 3-5 um thin sections. These sections were H&E counterstained following standard procedures outlined by Humason (1979, pp 118-120). If peripheral nerves were present, as in mammals, they would look like wavey bundles of tubes encased in fine connective tissue.

Gonads were prepared with HuC/D labels for developing neural tissue in the CNS and early PNS as well as elliptical germ cells (GCs) that appear green. Sox9 labels supportive (Sertoli) cells red. These cells dominate the testis. Laminin labels the basement membrane and shows as cyan. TuJ1 labels for neural processes reddish orange. Positive controls, controls with no primary antibody, and controls with no secondary antibody were run in red ear slider gonads from embryos incubated at female-promoting incubation temperatures and other at male-promoting incubation temperatures. Serial images of whole mounts were collected as series of optical Z-sections.
